# Hairy Cell Leukemia Diagnosed by Flow Cytometry Despite Absence of Hairy Cells in Peripheral Blood: A Case Report

**DOI:** 10.7759/cureus.88536

**Published:** 2025-07-22

**Authors:** Kotaro Nakano, Yusuke Fukatsu, Shinichiro Oka, Takumi Takahashi

**Affiliations:** 1 Hematology, Iwata City Hospital, Iwata, JPN

**Keywords:** flow cytometery, hairy cell leukemia (hcl), myelofibrosis, pancytopenia, peripheral blood smear, splenomegaly

## Abstract

Hairy cell leukemia (HCL) is a rare disease, and detecting hairy cells in peripheral blood is an essential initial step in its diagnosis. Flow cytometry (FCM) is a convenient and effective tool for assessing the immunophenotype, even without distinctive morphological features. We describe a case of HCL; primary myelofibrosis was initially suspected, but FCM findings ultimately led to the diagnosis of HCL. A 47-year-old male patient was admitted to the hospital with progression of pancytopenia, and splenomegaly was noted. The initial pathology report on bone marrow biopsy noted only MF-1 fibrosis. FCM examination showed positivity for CD19, CD20, CD11c, and CD25, and light-chain restriction. Bone marrow biopsy images showed fried egg-like cells, and *BRAF* V600E mutation was also positive, leading to the diagnosis of HCL. The patient was treated with cladribine and rituximab and achieved hematological recovery. This case highlights the usefulness of FCM for the diagnosis of HCL in the presence of splenomegaly, pancytopenia, and myelofibrosis, even though hairy cells may be absent in peripheral blood.

## Introduction

Hairy cell leukemia (HCL) is generally considered an indolent mature B-cell neoplasm and a rare disease, accounting for only 1.4% of all lymphomas [1]. The clinical features include splenomegaly and pancytopenia. Bone marrow fibrosis can be observed in cases of HCL [2]. Detecting hairy cells, leukemic cells with thin circumferential surface projections, in peripheral blood can be an important initial step in diagnosing HCL. A definitive diagnosis is established based on the immunophenotypic profile, with positivity for CD103, CD11c, CD25, CD123, Annexin A1, and the BRAF V600E mutant protein [3-5]. Flow cytometry (FCM) is a convenient and effective tool for assessing the immunophenotype, even in the absence of distinctive morphological features. In this report, we describe a case of HCL presenting with splenomegaly, pancytopenia, and bone marrow fibrosis, without hairy cells in peripheral blood. Primary myelofibrosis was initially suspected, but FCM findings ultimately led to the diagnosis of HCL.

## Case presentation

A 47-year-old male patient was admitted to the hospital with progression of pancytopenia. He had no specific complaints. The pancytopenia was incidentally identified on routine blood tests during a check-up conducted by the primary care physician. He had a history of hypertension and was taking amlodipine besylate and azilsartan. The patient also had panic disorder and was taking alprazolam.

**Figure 1:**
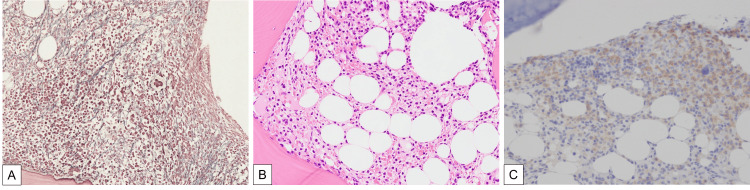
Bone marrow biopsy and aspiration showing (A) MF-1 bone marrow fibrosis with silver impregnation staining, (B) fried-egg cell, (C) leukemic cells expressing BRAF V600E mutant protein

**Figure 2:**
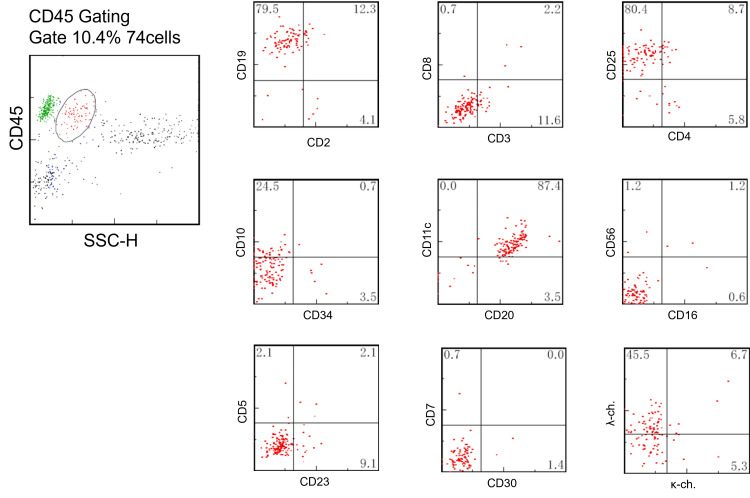
Flow cytometry results on bone marrow sample Lymphoid cells account for 10.4% and have positive expression of CD10, CD20, CD11c, CD25, and light chain restriction. SSC-H, side scatter height; CD, cluster of differentiation

Laboratory tests showed pancytopenia, no immature granulocytes, and no abnormal cells (Table 1). Computed tomography (CT) showed splenomegaly. The initial pathology report on bone marrow biopsy revealed MF-1 fibrosis (Figure 1A), but no abnormal cells were noted. FCM examination showed positivity for CD19, CD20, CD11c, and CD25, and light-chain restriction (Figure 2).

**Table 1:** Laboratory test results PT, prothrombin time; APTT, activated partial thromboplastin time; FDP, fibrinogen degradation product; sIL-2R, soluble interleukin-2 receptor

Test	Patient value	Units	Reference range
White Blood Cells	1100	/μL	3300-8600
Neutrophils	59.0	%	44.0-72.0
Lymphocytes	37.0	%	18.0-59.0
Monocytes	2.0	%	0.0-12.0
Eosionophils	1.0	%	0.0-10.0
Basophils	0.0	%	0.0-3.0
Red Blood Cells	322×10^4^	/μL	435-555×10^4^
Hemoglobin	11.2	g/dL	13.7-16.8
Hematocrit	32.7	%	40.7-50.1
Mean Corpuscular Volume	101.6	fl	83.6-98.2
Reticulocytes	2.47	%	0.2-2.5
Platelets	3.9×10^4^	/μL	15.8-34.8×10^4^
PT	10.6	sec	9.8-12.1
APTT	26.2	sec	24.0-34.0
Fibrinogen	305	mg/dL	200-400
FDP-D dimer	0.1	μg/mL	<=1.0
Sodium	140	mEq/L	138-145
Ptassium	3.4	mEq/L	3.6-4.8
Chloride	106	mEq/L	101-108
Calcium	9.1	mg/dL	8.8-10.1
Phosphate	2.3	mg/dL	2.7-4.6
Total Protein	7.8	g/dL	6.6-8.1
Albumin	4.9	g/dL	4.1-5.1
Total Bilirubin	1.2	mg/dL	0.4-1.5
Aspartate Aminotransferase	21	U/L	13-30
Alanine Aminotransferase	15	U/L	10-42
Lactate Dehydrogenase	197	U/L	124-222
Alkaline Phosphatase	109	U/L	38-113
Blood Urea Nitrogen	6	mg/dL	8.0-20
Creatinine	0.76	mg/dL	0.65-1.07
sIL-2R	8960	U/mL	157-474
C-Reactive Protein	0.15	mg/dL	0.00-0.14
Haptoglobin	97	mg/dL	30-200
Vitamin B12	182	pg/mL	180-914
Folate	12.3	ng/mL	>4.0

**Figure 3:**
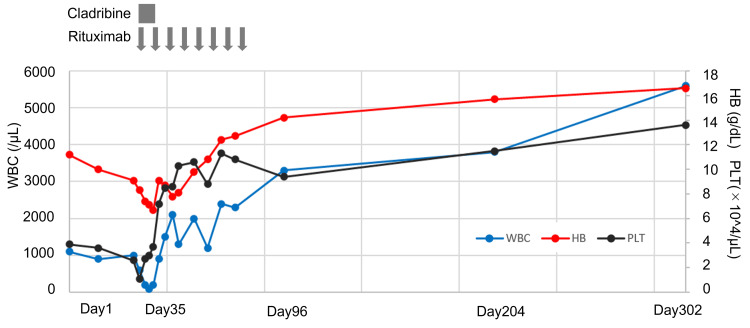
Clinical course WBC, white blood cells; HB, hemoglobin; PLT, platelets

Based on this, the patient was considered to have HCL. Bone marrow biopsy images were reviewed and showed fried egg-like cells (Figure 1B), and BRAF V600E mutant protein was also positive (Figure 1C), leading to a diagnosis of HCL. After the definitive diagnosis, a spontaneously dried specimen was prepared from peripheral blood, but no hairy cells were identified. The patient was treated with cladribine (0.09 mg/kg/day for seven days) and rituximab (administered weekly for eight cycles) [6]. As a result, the spleen size decreased, and hematological recovery was achieved (Figure 3). 

## Discussion

Identifying severe pancytopenia and careful observation of peripheral blood smears are the first steps in diagnosing HCL [2]. In the current patient, despite characteristic findings of HCL, such as pancytopenia and splenomegaly, it was not identifiable based on peripheral blood. The initial pathology report of the bone marrow biopsy only indicated MF-1 fibrosis. Morphologically, HCL was not apparent. While primary myelofibrosis was also considered, the diagnosis could not be reached without considering FCM with HCL as a differential diagnosis. Care needs to be taken in such cases; there is a report of a patient initially diagnosed with idiopathic myelofibrosis, who was re-diagnosed with HCL after treatment [7]. Careful observation of peripheral blood fractions is also useful in the differential diagnostic process. If there is no increase in juvenile granulocytes by extramedullary hematopoiesis, it suggests idiopathic myelofibrosis [8], and a review of the diagnosis may be considered. In the present case, there was no increase in juvenile granulocytes, and idiopathic myelofibrosis was the primary differential diagnosis prior to performing bone marrow examination, but it required careful consideration.

It is also important not to form a first impression based solely on findings from bone marrow puncture and biopsy of a patient presenting with pancytopenia and splenomegaly, but to again compare them with other clinical and blood-draw findings to determine if they are consistent with that diagnosis, if HCL is suspected. HCL is diagnosed by immunophenotype and expression of somatic mutations in *BRAF* V600E. In 98% of HCL patients, there are three or four cell-surface antigens: CD11c, CD103, CD123, and CD25. However, single or zero expression is normal in HCL-like disease [3]. In our case, FCM revealed a clonal population of B lymphocytes with dual expression of CD11c and CD25. Therefore, we considered this case as possible HCL. A histopathological review of the bone marrow biopsy specimen revealed a fried-egg image. Subsequently, a somatic mutation of *BRAF* V600E was identified, confirming the diagnosis. In the presence of symptoms suggestive of HCL, FCM testing with appropriate antibodies may be useful for diagnosis and differentiation.

There are patients in the early stages of the disease who do not present with pancytopenia, and there is no splenomegaly. FCM may be useful for diagnosis in such cases [9,10]. In our case, hairy cells were not detected in peripheral blood because the disease was in its early stages; it is possible that hairy cells would have been observed in more advanced stages of the disease. However, since the patient already had hypothrombocytosis, which is an indication for treatment, it was not possible to wait for hairy cells to become detectable in peripheral blood, and the FCM test was useful for early diagnosis.

Typical symptoms of HCL are pancytopenia and splenomegaly, but there are other rare manifestations. Symptoms caused by direct leukemic cell infiltration include skin changes, bone lesions, pulmonary changes, neurologic manifestations, ocular symptoms, hearing loss, and liver and gastrointestinal tract symptoms [11]. Careful FCM examination may be diagnostic even in patients presenting with these rare symptoms. When the cause is unclear, a review of peripheral blood smears and bone marrow may not be enough, and FCM studies in the setting of HCL may help facilitate an accurate diagnosis.

## Conclusions

In this case, we noted the following when encountering pancytopenia, splenomegaly, and myelofibrosis without any distinctive features in the peripheral blood. First, splenomegaly, pancytopenia, and myelofibrosis are not only key features of primary or secondary myelofibrosis associated with myeloproliferative neoplasms but also important characteristics of HCL. Second, when these features are present, careful examinations of peripheral blood smears and bone marrow are necessary to identify hairy cells or the fried-egg appearance. Finally, even in the absence of these features, FCM is an effective tool for identifying HCL.

## References

[1] Teras LR, DeSantis CE, Cerhan JR, Morton LM, Jemal A, Flowers CR (2016). 2016 US lymphoid malignancy statistics by World Health Organization subtypes. CA Cancer J Clin.

[2] Falini B, Tiacci E (2024). Hairy-cell leukemia. N Engl J Med.

[3] Matutes E, Morilla R, Owusu-Ankomah K, Houliham A, Meeus P, Catovsky D (1994). The immunophenotype of hairy cell leukemia (HCL). Proposal for a scoring system to distinguish HCL from B-cell disorders with hairy or villous lymphocytes. Leuk Lymphoma.

[4] Falini B, Tiacci E, Liso A (2004). Simple diagnostic assay for hairy cell leukaemia by immunocytochemical detection of annexin A1 (ANXA1). Lancet.

[5] Tiacci E, Schiavoni G, Forconi F (2012). Simple genetic diagnosis of hairy cell leukemia by sensitive detection of the BRAF-V600E mutation. Blood.

[6] Chihara D, Arons E, Stetler-Stevenson M (2020). Randomized phase II study of first-line cladribine with concurrent or delayed rituximab in patients with hairy cell leukemia. J Clin Oncol.

[7] Strupp C, Fenk R, Kündgen A, Gattermann N, Haas R, Germing U (2005). Hairy cell leukemia (HCL) with extensive myelofibrosis responds to thalidomide. Leuk Res.

[8] Mehta G, Rathod VM, Patel TM (2023). Primary myelofibrosis with extramedullary hematopoiesis - a case report with a review of literature. J App Hematol.

[9] Tadros J, Davis A, Awoleye O, Vassiliou E (2023). A case report of early diagnosis of asymptomatic hairy cell leukemia using flow cytometry. Front Immunol.

[10] Rahman K, Kumari S, Singh MK, Gupta R, Yadav G, Kumari N, Nityan S (2018). Atypical presentation of hairy cell leukemia: significance of CD200 on flow cytometry. J Cancer Res Ther.

[11] Robak T, Braun M, Janus A, Guminska A, Robak E (2024). Rare clinical symptoms in hairy cell leukemia: an overview. Cancers (Basel).

